# Minimally Invasive Techniques in Posterior Atlanto-Axial Fixation: State of the Art and Systematic Review

**DOI:** 10.3390/jcm14134657

**Published:** 2025-07-01

**Authors:** Gianpaolo Jannelli, Luca Paun, Cédric Y. Barrey, Paola Borrelli, Karl Schaller, Enrico Tessitore, Ivan Cabrilo

**Affiliations:** 1 Division of Neurosurgery, Department of Clinical Neurosciences, Geneva University Hospitals and Faculty of Medicine, University of Geneva, 1205 Geneva, Switzerland; 2 Department of Neurosurgery, Neurocenter of Southern Switzerland, Ente Ospedaliero Cantonale, 6900 Lugano, Switzerland; 3 Department of Spine and Spinal Cord Surgery, Neurological Hospital Pierre Wertheimer, GHE, Hospices Civils de Lyon, and Claude Bernard University Lyon 1, Bron, 69500 Lyon, France; 4 Department of Medical, Oral and Biotechnological Sciences, Laboratory of Biostatistics, University “G. d’Annunzio”, Chieti-Pescara, 66100 Chieti, Italy

**Keywords:** minimally invasive surgery, spine surgery, atlanto-axial, craniovertebral junction

## Abstract

**Background:** The atlanto-axial segment is highly mobile and, therefore, prone to instability in the setting of inflammatory disease, infection, tumor or trauma. While minimally invasive surgical (MIS) techniques have gained acceptance in the thoracolumbar spine due to their advantages over traditional approaches, their use at the atlanto-axial segment is controversial due to the surgical risk associated with its complex anatomy. To evaluate the current evidence on MIS atlanto-axial fixation, we carried out a systematic review of the literature and compared the reported results with those of open procedures. **Methods:** This systematic review follows PRISMA-DTA 2020 guidelines. A comprehensive search was conducted in November 2023 across PubMed/Medline, Google Scholar and clinicaltrials.gov using specific keywords related to minimally invasive atlanto-axial fixation. Data regarding study characteristics, patient demographics, surgical techniques, and outcomes were extracted from included studies. **Results:** This systematic review included 13 articles reporting on the results of surgery in 305 patients, in whom a total of 683 screws were inserted through a posterior MIS approach. N = 162 screws were inserted using the Harms–Goel technique, while N = 521 were placed using the Magerl technique. N = 40 screws were inserted using navigation guidance, while N = 643 were introduced with fluoroscopy assistance. Eight screws were misplaced. A Vertebral Artery (VA) injury was reported in three patients. With a mean value of 26.2 ± 15.3 months, the rate of fusion ranged between 80% and 100%. **Conclusions:** This study highlights the potential of MIS for posterior atlanto-axial fixation, which was achieved using Magerl transarticular screws in a large majority of cases. Despite technical challenges, MIS approaches appear to achieve satisfactory clinical and radiological outcomes with complication rates similar to those of open techniques. Future studies may help refine the indications for MIS and identify those cases better suited for open approaches.

## 1. Introduction

The atlanto-axial motion segment is the most flexible of the entire spine and this exceptional mobility predisposes it to instability [1,2]. It consists of two sets of joints: two lateral atlanto-axial joints, involving the inferior facets of the atlas’ lateral masses and the superior articular facets of the axis, and the median atlanto-axial joint, where the odontoid process forms a pivot around which the atlas rotates, stabilized by the transverse ligament [1,3,4,5].

Inflammatory disease, infection, tumor and trauma are all conditions that can affect this region of the spine [2,5,6,7,8,9,10]. When conservative treatment is not a management option, or when it fails, various surgical options are available, consisting of posterior or anterior fixations, or combined approaches [11]. With regard to the posterior approach, stabilization is traditionally achieved using either the Magerl or the Harms–Goel technique. In the Magerl technique, a posterior C1–C2 transarticular screw is inserted bilaterally from the C2 pars to the C1 anterior arch [12]. In contrast, the Harms–Goel technique involves a screw-and-rod-based fixation of the C1 lateral masses and the C2 pars or pedicles using polyaxial screws [13].

Minimally invasive surgical (MIS) technique has found increasing applications in the treatment of spinal pathologies, particularly in thoracolumbar procedures, due to its advantages over traditional open techniques in terms of reduced blood loss, muscle damage and postoperative pain, shorter surgical time and potentially faster recovery and hospital discharge [14,15,16,17,18,19].

The application of MIS principles to the craniovertebral junction, however, can be particularly challenging due to its complex anatomy, the risk of vertebral artery (VA) injury during screw placement, the lack of fusion surface and the steep surgical learning curve. Despite these difficulties, recent studies have investigated MIS approaches for posterior atlanto-axial fixation, using both the Magerl and Harms-Goel techniques, reporting favorable results in terms of safety as well as functional and radiological outcomes [20,21,22,23,24,25,26,27,28,29,30,31,32].

The goal of this systematic review is to report on the state of the art of MIS for atlanto-axial posterior fixations, focusing on five aspects: its feasibility and safety; its technical aspects; the impact of image guidance and navigation technologies in enabling MIS technique at the atlanto-axial level; its effect on intraoperative and hospitalization metrics; and the impact of reduced bony surface on fusion rates. 

## 2. Materials and Methods

The study protocol followed the Preferred Reporting Items for Systematic Reviews and Meta-Analyses (PRISMA-DTA) 2020 guidelines. We conducted a restricted search using the keywords “Percutaneous C1–C2”, “Percutaneous atlantoaxial”, “Minimally Invasive Atlantoaxial”, “Minimally invasive C1–C2” in November 2023 on the following databases: PubMed/Medline, Google Scholar and clinicaltrials.gov.

The first authors (GJ and LP) independently screened all titles and abstracts, and full-text copies of all relevant articles were obtained with exclusion of no pertinent studies. In case of discrepancy, the senior author (IC) arbitrated until a consensus among the authors was reached. The inclusion criteria were as follows: (1) Any study investigating the application of minimally invasive technique in posterior atlanto-axial fixations; (2) that includes at least 5 patients, which was set as an arbitrary threshold in an attempt to avoid the bias of results inherent to case reports and small case series; (3) and written in English. The exclusion criteria were therefore the following: (1) studies involving less than 5 patients; (2) studies written in languages other than English. A broad literature search was intentionally conducted to minimize the risk of identifying too few eligible studies, which could otherwise have generated a selection bias due to the small number of reported patients.

### 2.1. Risk of Bias and Quality of Studies

The articles that met the inclusion criteria were independently graded by two authors (GJ and LP) according to the Newcastle–Ottawa Quality Assessment Scale for quality assessment of non-randomized studies. The senior author (IC) was available to resolve disagreements. The level of evidence for each study was evaluated using the Oxford Centre for Evidence-Based Medicine guidelines.

### 2.2. Data Collection

Two of the authors (LP and GJ) extracted the data independently, with the senior author (IC) available to resolve disagreements. The following items were included: (1) Study ID; (2) study characteristics (authors, year, country where the study was performed, type of study); (3) patient demographics; (4) sample size; (5) diagnosis for surgical indication; (6) number of screws; (7) type of surgical approach; (8) navigation or fluoroscopy assistance; (9) number of misplaced screws; (10) fusion achievement at radiological follow-up; (11) type of fusion performed intraoperatively (autograft versus allograft); (12) cases of VA injury; (13) duration of surgery; (14) blood loss; (15) postoperative pain levels; (16) hospital length-of-stay (H-LoS); (17) duration of follow-up.

### 2.3. Statistical Analysis

Descriptive statistics were reported as means and standard deviations (SD) for the quantitative variables, and as absolute values and percentages for qualitative variables. Differences in quantitative variables between the two techniques (Magerl versus Harms) were evaluated with the Student’s *t*-test for independent data. A *p*-value < 0.05 was considered statistically significant. All analyses were performed using Stata software, version 18.0 MP (StataCorp, College Station, TX, USA).

## 3. Results

**Figure 1:**
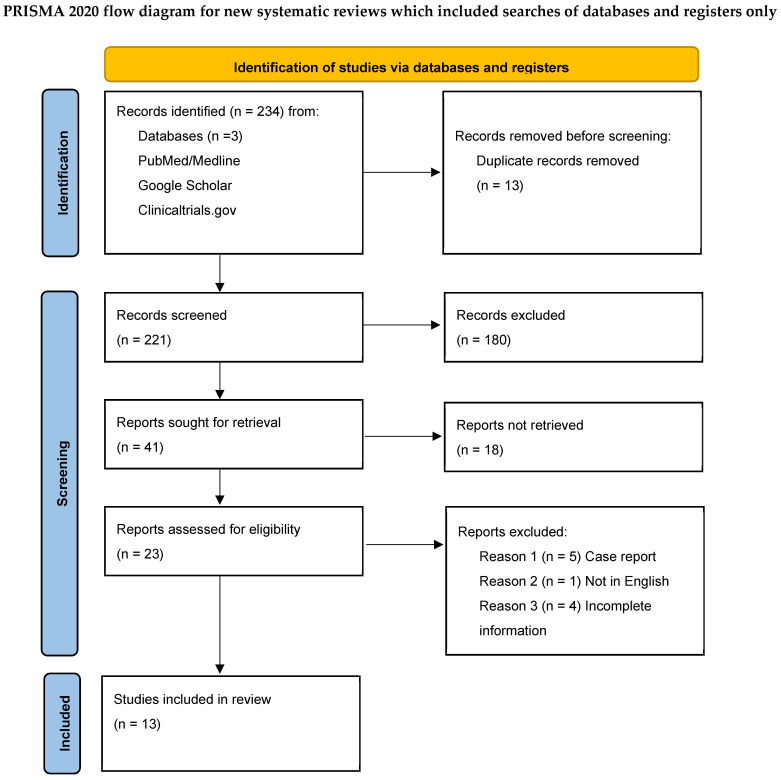
PRISMA-P 2020 flow-chart and search strategy.

The literature search yielded 234 articles (Figure 1). After removing duplicates, the abstracts of 221 articles were screened, of which 180 were irrelevant and were therefore excluded. After full-text review of the remaining 41 references, 13 were ultimately included (reasons for exclusion during full-text screening are reported in Figure 1). The quality assessment of the included studies is reported in Table 1 using the Newcastle–Ottawa scale. Due to the heterogeneity in these studies’ methodology and of their reported outcomes, a quantitative data analysis was not feasible.

A total of 305 patients were included. N = 73 patients were male, N = 129 were female and sex was not specified in the remaining N = 103. Mean age was 59.7 ± 14.4 years.

N = 149 presented with a history of trauma. N = 71 were diagnosed with a type II dens fracture according to Anderson–D’Alonzo, seven with a type III Anderson–D’Alonzo fracture and N = 31 underwent surgery due to non-union following conservative treatment. N = 83 patients underwent surgery due to rheumatoid arthritis involvement of the atlanto-axial joint. Remaining diagnoses and demographic data are comprehensively reported in Table 2.

A total of 683 screws were inserted through a posterior MIS approach. N = 162 screws were inserted using the Harms–Goel technique, while N = 521 were placed using the Magerl technique. N = 40 screws were inserted using navigation guidance, while N = 643 were placed with fluoroscopy assistance. Eight screws were misplaced. A VA injury was reported in three patients. N = 545 screws were inserted percutaneously, with endoscopic assistance for 14 of these. The remaining 138 screws were inserted through the transmuscular approach, using tubular retractors in N = 108 screws and endoscopic assistance in 30 screws.

Data concerning the duration of surgery was available for 214 patients. The mean value calculated for this sample was 135.7 ± 58.8 min.

An interarticular allograft was used in N = 29 patients, while an interarticular autograft was used in three patients. In N = 121 cases, a bone autograft was fixed with Gallie technique. Both interlaminar and interarticular graft were placed in N = 82 cases.

Data concerning follow-up were available in ten studies for a total of 279 patients, with a mean value of 26.2 ± 15.3 months. In nine studies, the rate of fusion ranged between 80% and 100%. Only one study reported a rate of fusion of 5% (at 6 months follow-up). In four studies, fusion was assessed with a CT, in two studies by radiography, in two studies by both techniques. Two studies do not specify what exam was performed to evaluate the fusion. A summary of the results is shown in Table 3. 

When comparing the Magerl and Harms–Goel techniques, operative time and blood loss were significantly lower in the Magerl technique group (*p* < 0.001 and *p* = 0.005, respectively). Conversely, patient age and H-LoS were significantly higher in the Magerl technique group (p = 0.039 and *p* < 0.001, respectively), as summarized in Table 4.

A meta-analysis of postoperative outcomes was attempted, but did not yield meaningful or statistically significant results due to high heterogeneity among the studies and the small number of included patients.

## 4. Discussion

Minimally invasive techniques are becoming increasingly prevalent in spine surgery. Relevant literature demonstrates that the percutaneous and Wiltse approaches, when feasible, are associated with reduced soft tissue disruption, reduced destabilization of spinal segments, enhanced recovery after surgery (ERAS) and lower infection rates compared to open procedures [14,18,19,33,34,35].

However, MIS techniques have mainly found applications in thoracolumbar pathologies. Little is known about the applicability and the impact of MIS in the cervical spine, and even less on the C1–C2 segment. Indeed, to the best of our knowledge, this is the first systematic review to investigate the application of MIS technique in posterior atlanto-axial fixation. Where feasible, we compare the outcomes of the MIS technique with those reported for the open Harms–Goel technique in a recent systematic review with meta-analysis [36].

### 4.1. Safety and Feasibility of MIS C1–C2 Posterior Fixation

The complex anatomy and the presence of vital neurovascular structures are a challenge to performing surgery at the C1–C2 level and require meticulous intraoperative technique to mitigate the risks of severe morbidity and mortality. A thorough analysis of preoperative imaging studies and expert knowledge of standard anatomic landmarks are essential, even for open procedures. VA anomalies must be ruled out by a preoperative angio-CT. In MIS approaches in particular, the lack of a comprehensive intraoperative view of the anatomy can be disorienting to the surgeon, representing a primary limitation of the application of MIS to the atlanto-axial segment.

In our review, including 305 patients with C1–C2 MIS posterior fixation, a total of 683 screws were placed, of which 8 were misplaced (1.2%). This value is inferior to the 5.8% rate of screw misplacement in open surgeries [36].

There were no spinal cord injuries. Three cases were complicated with a VA injury (1%). This rate is lower than that reported for open procedures. Indeed, a recent meta-analysis found the risk of VA injury with the Harms technique to be 2.8% [36]. It is slightly higher with the Magerl technique, varying from 0% to 10% in different series [37,38,39,40].

Pre-study predictions for MIS C1–C2 posterior fixations would likely have anticipated, at best, rates comparable to those with open procedures. The apparent superiority in screw accuracy and safety observed in the MIS cohort may be related to the likely selective application of this novel technique to patients with favorable—and therefore less “risky”—anatomy. Additionally, MIS C1–C2 fixations are more likely to be performed by surgeons with experience with craniovertebral junction anatomy. Finally, our MIS cohort is also numerically smaller than the patient groups in meta-analyses of open procedures and so may fail to capture the true complication rate.

Two studies reported on endoscope-assisted screw insertion [24,25,26]. According to their findings, the use of the endoscope may be helpful in identifying the correct screw entry-point for both Magerl and Harms techniques, improving procedural safety and accuracy.

### 4.2. Technical Aspects of MIS C1–C2 Posterior Fixation

Of the 683 screws placed using MIS, N = 162 screws were inserted using the Harms-Goel technique, while the greater part—N = 521—were placed using the Magerl technique. Although both techniques provide similar results in terms of stability and patient satisfaction, the Harms-Goel technique is reported to be safer and associated with fewer complications [36,37,38,39,40,41]. And yet, as exemplified in this systematic review, the Magerl technique may be better suited in the setting of MIS, possibly due to the fact that it solely relies on the insertion of stand-alone transarticular C1–C2 screws. This is in contrast with the Harms-Goel technique that requires placing a rod to connect the C1 and C2 screwheads. Rod placement and tightening through a narrow surgical corridor to the C1–C2 region may not only be technically difficult but potentially dangerous.

Other aspects of C1 lateral mass screw insertion, not readily performed through an MIS approach—such as inferiorly displacing the C2 nerve root with a dissector in order to avoid injuring the nerve root during screw insertion, or the frequent need to nibble away bony overhang off the posterior arch of C1 to allow access to the screw entry-point in the upper aspect of the lateral mass—may represent further reasons to favor the Magerl technique over the Harms technique in MIS C1–C2 posterior fixations [42,43,44].

### 4.3. Image Guidance and Navigation in MIS C1–C2 Posterior Fixation

Regarding the use of image guidance during these procedures, our review found that only N = 40 screws were inserted using navigation, while N = 643 were placed under fluoroscopy. These results contrast with current literature that advocates the use of navigation for atlanto-axial instrumentation due to the reported lower risk of screw misplacement and VA injury [5,45,46,47,48]. The overwhelming use of fluoroscopy over navigation observed in this review may arise from the fact that most of the included reports predate some of the initial reports of navigation in the axial cervical spine [49,50]. Furthermore, not all surgical centers are equipped with a navigation system [51]. Additionally, the accuracy of navigation systems may be perceived as insufficiently reliable for the high-risk anatomy of the craniovertebral junction [52]. Indeed, mobility of the C1–C2 segment may contribute to reduced navigation accuracy in this region, and its sleeker anatomy, compared to that of the lumbar spine, may be less tolerant of imprecision. Furthermore, the irregular anatomy of the atlanto-axial segment, the paucity of reliable external bony landmarks compared to thoracolumbar vertebrae, the narrow surgical corridor, and the deep surgical field can pose challenges for navigation, potentially leading to registration difficulties and reduced accuracy in posterior C1–C2 surgery. Nevertheless, recent evidence suggests that navigation-assisted C1–C2 fixation can achieve high levels of accuracy despite these anatomical and technical limitations [53,54].

Finally, attaching the navigation reference star to the unexposed C2 spinous process is impractical in a percutaneous MIS approach. That said, securing the reference star to the Mayfield head-holder has been shown to be a reliable alternative in C1–C2 posterior fixation [5] and may therefore be a suitable solution for MIS procedures.

### 4.4. Effects of MIS C1–C2 Fixation on Intraoperative Metrics and Postoperative Hospitalization

Our review shows that MIS atlanto-axial fixation is associated with a mean duration of surgery of 135.7 ± 58.8 min, a mean blood loss of 158.1 ± 150.2 mL and a mean hospital-length of stay of 6.7 ± 4.4 days. In their systematic review with meta-analysis on the efficacy and safety of the open Harms-Goel technique, Lvov et al. [36] reported a mean operative time and mean blood loss of 144 min and 264 mL, respectively. The reduced blood loss observed in MIS can be explained by the net reduction in muscle dissection and reduced, or even absent, venous plexus manipulation during screw insertion.

Tanenbaum et al. [55] investigated trends and predictors of outcomes and hospital costs following open atlanto-axial fusion, reporting a median hospital-length of stay of 6 days, similar to our findings [55]. The authors also observed that older patients with more comorbidities had higher in-hospital mortality rates, longer hospital stays and greater overall mean hospitalization costs. These findings highlight the potential value of applying MIS principles in this particularly fragile patient population.

### 4.5. Fusion Rate

Our review identified a mean fusion rate of 84%, which is slightly lower than the 95.8% fusion rate reported in recent literature for the open approach at 24 months’ time. [36] This said, all studies included in our analysis reported fusion rates between 80% and 100%, with the exception of a single series documenting a rate of only 5% [21]. Notably, the latter study had a mean follow-up of six months, whereas the other studies reported follow-up durations ranging from 11 to 32 months. Given that cervical spine fusion generally starts to occur after 9–12 months, with rates increasing over time [56,57], it is plausible that a longer follow-up in this cohort would have shown a higher fusion rate.

In four studies, fusion was performed using allograft, in three studies using autograft, and one study used both [20,22,23,24,28,30,31,32]. An interarticular allograft was used in N = 29 patients, while an interarticular autograft was used in three patients. In N = 121 cases, a bone autograft was fixed with Gallie technique. Both interlaminar and interarticular graft were placed in N = 82 cases. Older studies mainly used autograft, whereas more recent ones favored allograft. Historically, iliac crest autograft was the standard of care for bone grafting but was associated with longer operating times, greater blood loss, postoperative gait difficulties, increased infection risk and donor site pain [48]. Recently, Zhang et al. [58], Godzik et al. [59] and Iyer et al. [60] compared autograft and allograft in atlanto-axial screw-and-rod fixation, reporting similar fusion rates—approaching 100%—for both, including in the pediatric population where higher complications were observed with autograft, however.

Finally, no graft was used in five studies included in our review [21,25,26,27,29]. Nonetheless, their fusion rates were comparable to those of the other studies in our review that had utilized grafting, with the exception of Koepke et al. [21], where the short postoperative follow-up accounted for the low fusion rate, as discussed above.

### 4.6. Future Perspectives

Although the use of MIS remains limited to select cases, advancements in technology, such as modern diagnostic imaging with high-resolution preoperative three-dimensional planning, customized implants, navigation, artificial intelligence and robotics, have the potential to address the complexity of craniovertebral junction anatomy, facilitating the adoption of MIS even in this region [48,61,62,63,64,65,66,67].

Artificial intelligence in spine surgery may offer advantages for planning surgeries and assisting with precision maneuvers and intraprocedural decision-making [61]. Customized 3D-printed bone grafts [63,64] could be individually tailored to the specificities of the MIS approach, to offer the possibility for bone graft placement despite the limitations of reduced fusion surface.

Finally, although their current applications are primarily in the thoraco-lumbar spine, robotic assistance and augmented reality have been reported as reliable and safe in cervical spine procedures, and therefore appear well-suited to support the transition towards minimal invasiveness in C1–C2 posterior fixation [65,66,67]. One technique-specific advantage, however, of augmented reality-based navigation over robotic systems is the ability for the user to visually verify the system’s accuracy through overlay of the virtual segmentations on the real-world anatomy; this benefit, however, would be limited in minimally invasive cervical procedures due to the paucity of internal anatomical landmarks with which to confront the augmented surgical field [48,68].

Furthermore, recent literature supports the implementation of enhanced recovery after surgery (ERAS) protocols in posterior cervical procedures, demonstrating significant benefits in terms of length of hospital stay, return of physiological function, complications and pain scores [69]. The principles of MIS align with these objectives, and its integration into surgical practice may represent an advancement in the management of atlanto-axial instability, particularly in elderly patients with multiple comorbidities.

### 4.7. Limitations

Aside from the data heterogeneity of the included studies, several articles reported only small patient series, which posed an additional limitation for conducting a meta-analysis.

The review included only retrospective studies, and the absence of randomized trials limits the strength and generalizability of the findings.

Patient selection bias must also be considered, as MIS posterior atlanto-axial fixation is most likely reserved for patients with favorable anatomy and carried out by experienced surgeons.

Pain outcomes were inconsistently reported across studies. When present, they were assessed using different scales (e.g., VAS, NDI), which complicated a comprehensive evaluation of the clinical benefits of minimally invasive approaches.

Our review included all patients treated with MIS posterior C1–C2 fixation, irrespective of the underlying pathology (e.g., trauma, infection, degenerative disease, tumor). Consequently, clinicoradiological outcomes may vary based on the specific diagnosis.

Moreover, the term “minimally invasive” in this systematic review is used broadly to define a range of different approaches that serve as alternatives to the traditional open technique, including purely percutaneous, tubular-assisted, and endoscopic-assisted methods. Current evidence does not support superiority of one MIS technique over another.

Lastly, a consistent analysis of fusion rates was limited by variability in follow-up durations and in the methods used for radiological assessment of fusion.

## 5. Conclusions

This systematic review highlights the potential of MIS for posterior atlanto-axial fixation. Despite the technical challenges inherent to the craniovertebral junction, MIS approaches appear to achieve satisfactory clinical and radiological outcomes with complication rates at least comparable to traditional open techniques. Future research will likely optimize MIS technique for C1–C2 posterior fixation by refining the indications best suited for MIS versus those better managed through open surgery.

## Figures and Tables

**Table 1:** Newcastle–Ottawa scale (NOS) assessment of non-randomized studies.

Study	Selection (0–4)	Comparability (0–2)	Outcome (0–3)	Total (0–9)
Gelinne et al., 2023 [20]	4	2	3	9
Koepke et al., 2022 [21]	3	1	2	6
Kaminski et al., 2008 [22]	3	2	3	8
Schmidt et al., 2006 [23]	3	2	3	8
ElSaghir et al., 2005 [24]	3	1	2	6
Shi et al., 2020 [25]	3	2	2	7
Meyer et al., 2020 [26]	4	2	3	9
Lvov et al., 2019 [27]	4	2	3	9
Dusad et al., 2018 [28]	4	2	3	9
Alhashash et al., 2018 [29]	4	2	2	8
Srikantha et al., 2016 [30]	3	1	2	6
Díaz et al., 2014 [31]	3	1	2	6
Holly et al., 2010 [32]	3	2	2	7

**Table 2:** Patient demographics, surgical variables and clinical outcomes.

Year, Author	Sample Size (n)	Gender (n, M/F)	Mean Age (Years)	Diagnosis (Type, n)	Screw (n)	Technique	Approach Type	Screw Misplacement (n)	Operative Time (Min ± SD)	Rate of Fusion (%)	Fusion	Blood Loss (mL ± SD)	H-LoS (Days ± SD)	Post Operative Follow-Up (Months)	Postoperative Pain (n ± SD, Scale)
**2023, Gelinne** **et al.** [20]	5	3/2	70	Trauma, 5	20	Harms	Percutaneous	0	234	NA	Interarticular cages with allograft	30	2	NA	7 (VAS)
**2022, Koepke** **et al.** [21]	23	17/6	73	Trauma, 19 Malignancy, 3 Autoimmune, 1	46	Magerl	Percutaneous	2	NA	5	No	NA	10 ± 5.68	6	2.6 ± 2.5 (VAS)
**2020,** **Shi** **et al.** [25]	7	5/2	73	Trauma, 7	14	Magerl	Endoscopic assisted percutaneous unilateral	0	131.1	100	No	<50	NA	16.9	16.9 (NDI)
**2020, Meyer** **et al.** [26]	5	NA	NA	Trauma, 5	20	Harms	Percutaneous	0	NA	80	No	NA	4	11.2	NA
**2019,** **Lvov** **et al.** [27]	15	12/3	44	Trauma, 15	30	Magerl	Endoscopic assisted Transmuscular	0	90	90	No	50	NA	58	1 (VAS)
**2018, Dusad** **et al.** [28]	82	NA	36.26	Trauma, 54 Autoimmune, 9 Infectious, 8 Hypoplastic, 7 Osteoarthritis, 5 Syndromic, 4	163	Magerl	Percutaneous	0	120.11 ± 15.82	97.5	Allograft (interlaminar and interarticular)	104.84 ± 21.75	7	24	3.3 ± 1.12 (VAS)
**2018,** **Alhashash et al.** [29]	20	11/9	81	Trauma, 20	40	Magerl	Percutaneous	0	51.75 ± 13.7	88	No	41.7 ± 31.57	14.15 ± 4.48	22.28	2.4 (VAS)
**2016,** **Srikantha et al.** [30]	5	3/2	45	Instability, 3 Trauma, 2	20	Harms	Transmuscolar Tubular assisted	1	192	80	3 Autograft 2 Allograft (interarticular)	260	7.4	19	NA
**2014,** **Diaz** **et al.** [31]	16	NA	57.5	Trauma, 8 Autoimmune, 8	64	Harms	Transmuscular Tubular assisted	0	193.7	NA	Allograft (interarticular)	404	2	NA	NA
**2010,** **Holly** **et al.** [32]	6	5/1	51	Trauma, 5 Os Odontoideum, 1	24	Harms	Transmuscolar Tubular assisted	0	NA	100	Allograft (inter articular)	100	NA	32	NA
**2008,** **Kaminski et al.** [22]	47	19/28	74.9	Trauma, 28	94	Magerl	Percutaneous	3	98	100	Autograft (Gallie)	NA	NA	42	NA
**2006, Schmidt** **et al.** [23]	17	12/5	53.4	Trauma, 9 Autoimmune, 8	34	Magerl	Percutaneous	0	110.6 ± 23.7	NA	Autograft (Gallie)	382.6 ± 406.2	NA	NA	NA
**2005, Elsaghir** **et al.** [24]	57	3/54	57	Autoimmune, 57	114	Magerl	Percutaneous	1	NA	98	Autograft (Gallie)	NA	NA	30.4	NA

Acronyms and abbreviations: n—Number; M/F—Male/Female; SD—Standard Deviation; VAS—Visual Analog Scale; NDI—Neck Disability Index; CT—Computed Tomography; H-LoS—Hospital-Length of Stay; NA—Not Available/Not Applicable; X-ray—Radiographic Imaging (Plain Film).

**Table 3:** Main population characteristics.

Patients (n)	305
**Age (years)**	59.7 ± 14.4
**Technique**	
***Harms–Goel***	37 (12.1)
***Magerl***	268 (87.9)
**Fusion rate**	84%
**Blood loss (ml)**	158.1 ± 150.2
**VA injury (n, %)**	3, 0.9
**Length of surgery (minutes)**	135.7 ± 58.8
**H-LoS (days)**	6.7 ± 4.4
**Follow-up (months)**	26.2 ± 15.3

Data are expressed as means ± SD or n (%) where indicated. n: Number. VA: Vertebral artery. H-LoS: Hospital-Length of stay.

**Table 4:** Comparison between Magerl and Harms–Goel techniques in C1–C2 fixation.

	Magerl (N = 268)	Harms–Goel (N = 37)	*p*-Value
**Age (years)**	61.6 ± 16.3	55.9 ± 10.7	**0.039**
**Operative time (min)**	100.3 ± 28	206.6 ± 23.8	**<0.001**
**Fusion rate (%)**	82.6 ± 34.6	86.7 ± 11.6	0.475
**H-LoS (days)**	9.1 ± 4.4	3.9 ± 2.6	**<0.001**
**Blood loss (ml)**	125.8 ± 145.7	198.50 ± 167.4	**0.005**

Data are expressed as mean ± SD; *p*-values are for Student’s *t*-test for independent data (in bold the significant differences). H-LoS: Hospital length-of-stay.

## Data Availability

All data are available in the text.

## Abbreviations

MIS	Minimally Invasive Surgery
VA	Vertebral Artery
H-LoS	Hospital Length-of-Stay
SD	Standard Deviation
VAS	Visual Analog Scale
NDI	Neck Disability Index
CT	Computed Tomography
NA	Not available/Not applicable
ERAS	Enhanced Recovery After Surgery
